# Emerging roles of TFE3 in metabolic regulation

**DOI:** 10.1038/s41420-023-01395-0

**Published:** 2023-03-11

**Authors:** Xingyu Li, Yongming Chen, Siqiao Gong, Huixia Chen, Huafeng Liu, Xiaoyu Li, Junfeng Hao

**Affiliations:** Institute of Nephrology, and Guangdong Provincial Key Laboratory of Autophagy and Major Chronic Non-Communicable Diseases, Affiliated Hospital of Guangdong Medical University, Zhanjiang, 524001 China

**Keywords:** Autophagy, Cell growth, Metabolic disorders, Metabolism

## Abstract

TFE3 is a member of the MiT family of the bHLH-leucine zipper transcription factor. We previously focused on the role of TFE3 in autophagy and cancer. Recently, an increasing number of studies have revealed that TFE3 plays an important role in metabolic regulation. TFE3 participates in the metabolism of energy in the body by regulating pathways such as glucose and lipid metabolism, mitochondrial metabolism, and autophagy. This review summarizes and discusses the specific regulatory mechanisms of TFE3 in metabolism. We determined both the direct regulation of TFE3 on metabolically active cells, such as hepatocytes and skeletal muscle cells, and the indirect regulation of TFE3 through mitochondrial quality control and the autophagy–lysosome pathway. The role of TFE3 in tumor cell metabolism is also summarized in this review. Understanding the diverse roles of TFE3 in metabolic processes can provide new avenues for the treatment of some metabolism-related disorders.

## Facts

1. The reduction or absence of TFE3 is an underlying mechanism of energy metabolism disorders in organisms.
2. TFE3 can directly regulate cellular metabolism, as well as through the regulation of mitochondrial, lysosome, and autophagy activity.
3. Screening effective interventions for TFE3 to regulate body energy metabolism should be the focus of future research.

## Open questions

1. What is the mechanism of TFE3 regulation of lipid metabolism? Will excessive TFE3 expression inhibit lipid metabolism?
2. Is there a negative feedback regulation between TFE3 and the mTOR signaling pathway? What are the mechanisms that provoke negative feedback regulation?
3. Can drugs that directly or indirectly regulate TFE3 treat clinical metabolic diseases?

## Introduction

Metabolism is one of the most basic features of life and is an initiating factor of cellular life activity. Numerous pathways are conserved during the evolution of animals, plants, fungi, and bacteria [1, 2]. Several factors, such as abnormal levels of glucose and lipid metabolism, altered mitochondrial dynamics, and changes in autophagy activity, can affect metabolism. TFE3, an evolutionarily conserved transcription factor, is involved in the regulation of these anomalous events [1, 3–5].

Three of the four MiT family members, TFE3, TFEB, and MITF, have been established to regulate metabolism, and only TFEC has been found to be unrelated to metabolism. TFEB regulates lipid metabolism in liver cells by regulating the transcription of peroxisome proliferator-activated receptor-gamma coactivator 1 (PGC-1α), a key regulator of energy metabolism. TFEB also controls glucose homeostasis and energy balance in skeletal muscle [6–9]. MITF is a positive regulator of PGC-1α [10]. In melanocytes, MITF promotes silencing information regulator 2 (SIR2)-related enzyme 1 gene expression, which is the major metabolic sensor in cells that deacetylates and suppresses the key lysine acetyltransferase p300 [11, 12]. TFE3 is universally expressed, particularly in metabolically vigorous organs, such as the liver and muscle. The liver is a key organ regulating glucose and lipid metabolism in vivo, and the loss of TFE3 leads to glucose and lipid metabolism disorders in the liver [4]. Skeletal muscle can respond to the increased energy demands of muscle contraction by regulating metabolism; however, muscle contraction exercise promotes TFE3 nuclear translocation and activation in mouse skeletal muscle, through the Ca^2+^-stimulated protein phosphatase calcineurin [13].

TFE3 generally forms a homodimer with itself or a heterodimer with TFEB. The dimer subsequently enters the nucleus to perform various biological functions [14]. The most studied functions of TFE3 include lysosomal biogenesis promotion, involvement in cancer progression, and autophagy induction [5, 15]. Recent studies have revealed that *Tfe3* knockout (KO) mice exhibit abnormal mitochondrial metabolism, abnormalities in systemic glucose and lipid metabolism, and an increased risk of obesity and diabetes induced by a high-fat diet. In these cases, the overexpression of TFEB can rescue part of the phenotype [4]. These results suggest that the cooperation of TFE3 with TFEB plays a critical role in regulating metabolic processes in the body. However, the mechanism through which TFE3 regulates energy metabolism has not yet been systematically elucidated.

In this review, we highlight the latest findings regarding the mechanisms of TFE3 in regulating metabolism, which may allow a better understanding of the role of TFE3 in related metabolic diseases and offer a prospective summary of the application of TFE3 as a potential treatment target for metabolic diseases.

## TFE3 directly regulates the metabolism of hepatocytes, skeletal muscle cells, adipocytes, and tumor cells

### TFE3 plays an important role in the regulation of glucose and lipid metabolism in the body

TFE3 is a bHLH transcription factor that binds to E-box sequences [16]. E-box is a gene segment that is involved in several metabolism-related processes, such as glycolysis, insulin signaling, and lipid metabolism [17–19]. This suggests that TFE3 may be involved in glucose and lipid metabolism. To explore the effect of TFE3 on metabolism, Pastore et al. simultaneously fed *Tfe3* KO and wild-type (WT) mice a high-fat diet and found significantly high body fat content in *Tfe3* KO mice. Indirect calorimetry revealed a significant increase in the respiratory exchange rate in *Tfe3* KO mice, indicating preferential utilization of carbohydrates and reduced lipid catabolism. They also identified reduced energy expenditure in *Tfe3* KO mice, which may provide an explanation for the obesity phenotype in the species. To further understand the effects of TFE3 on metabolism, they used adenovirus-expressing human TFE3 to achieve TFE3 overexpression in the liver of WT mice on a high-fat diet and found that the body weight, fatty liver, and glucose tolerance of these mice improved. Both the knockdown and overexpression of TFE3 showed that it plays a pivotal role in glucose and lipid metabolism [4].

### TFE3 can regulate the daily metabolism of hepatocytes by regulating insulin and glucose homeostasis

**Fig. 1:**
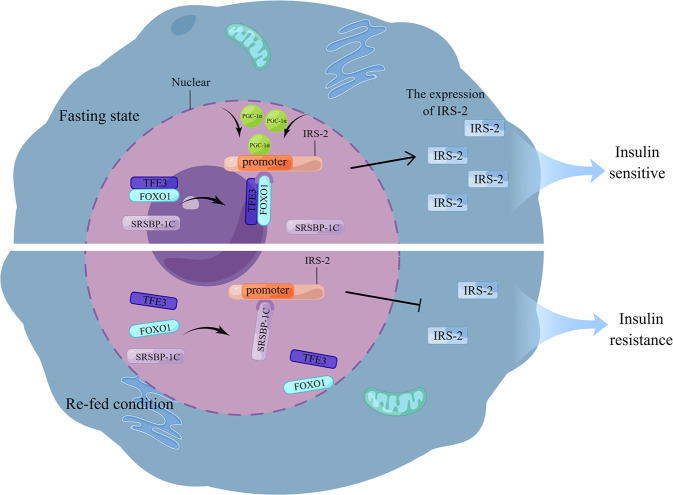
Schematic of TFE3 and SREBP-1C regulating hepatocyte sensitivity to insulin. In the fasting state, TFE3 and FOXO1 interact and bind to the IRS-2 promoter and co-activate IRS-2 through PGC-1α, recruited by SRSBP-1C, to increase IRS-2 expression and insulin sensitivity. In the re-fed condition, activated SRSBP-1c will competitively bind IRS-2, inhibit IRS-2 expression, and produce insulin resistance.

Insulin plays an important role in metabolism, and the role of the insulin receptor substrate (IRS) protein cannot be ignored. IRS proteins, primarily IRS-2, can bind to insulin receptors and perform a biological role [20, 21]. Forkhead box O 1 (FOXO1) and sterol regulatory element binding protein-1c (SREBP-1c) competitively bind to the *IRE/SRE* site in the human *IRS-2* gene. FOXO1 causes IRS-2 activation, whereas SREBP-1c causes IRS-2 inhibition. In the fasting state, plasma insulin levels are low. Both TFE3 and FOXO1 physically interact and then co-bind to the promoter of IRS2, thereby transactivating IRS-2 expression. The implementation of this process may also depend on SREBP-1c inactivation, leading to the recruitment of co-activator PGC-1α [22, 23]. When hepatocytes are re-fed, SREBP-1c can seize the binding site between TFE3/FOXO1 and IRS-2, inhibiting the expression of IRS-2 and causing hepatic cell insulin resistance (Fig. 1). In conclusion, SREBP-1c, FOXO1, and TFE3 can control hepatocyte sensitivity to insulin by regulating IRS-2 expression [18, 22]. Simultaneously, TFE3 can promote the expression of insulin-induced gene 1, which prevents SREBP/SREBP cleavage-activating proteins in the rough endoplasmic reticulum (ER) from entering the Golgi apparatus, thereby inhibiting SREBP activity. This explains the unusual phenomenon that although TFE3 increases hepatocyte sensitivity to insulin, lipid synthesis does not increase [24].

TFE3 reduces blood glucose levels in diabetic mice by increasing hepatocyte sensitivity to insulin and in Type 1 diabetic mice through streptozotocin-induced insulin depletion. This also suggests that TFE3 can activate insulin signaling via other mechanisms, in addition to increasing the signaling levels of the IRS-2/Akt pathway [18]. Following TFE3 overexpression in rat primary hepatocytes, Shimano et al. found that TFE3 enhanced the phosphorylation level of GSK3 β, a key regulator of glycogen synthesis, even in the absence of insulin. In addition, other insulin signaling pathways, such as the extracellular signal-regulated kinase pathway, can be activated by TFE3. Therefore, the intensity of TFE3 activation in the entire insulin signaling pathway considerably exceeds that of real insulin supplementation [18, 24]. For metabolic disorders caused by insufficient insulin function, it may be possible to use a method that directly or indirectly improves TFE3 activity to assist in the treatment of such disorders.

TFE3 interacts with the molecules of the insulin-related pathway to regulate metabolism and glucose homeostasis. Promoter analysis revealed the overexpression of TFE3 could also enhance the expression of hexokinase 2 (*HKII*) and liver-type glucokinase in hepatocytes. Simultaneously, some studies revealed that these two genes were downstream targets of TFE3 [18, 25]. The nuclear receptor, REV-ERBα, is involved in lipid, bile acid, and glucose metabolism in the liver. The administration of REV-ERBα agonizts to diet-induced obese mice significantly alleviates their blood lipid, blood glucose, and obesity phenotypes [26]. However, in REV-ERBα-deficient mice, the liver undergoes steatosis, which affects the synthesis and fecal excretion of bile acids [27, 28]. When REV-ERBα is knocked down in human hepatoma carcinoma cells, the expression of glucose-6-phosphatase and phosphoenolpyruvate carboxykinase, which encode glycogen synthase, is significantly increased [29]. Simultaneously, TFE3 can reportedly bind to the REV-ERBα promoter [30]. Thus, TFE3 can inhibit adipose tissue and glycogen genesis by promoting REV-ERBα expression and enhancing bile acid metabolism.

### TFE3 can promote glucose metabolism levels in skeletal muscle cells

In addition to the liver, skeletal muscle is a target organ for insulin. Skeletal muscle can uptake, consume, and store a portion of insulin-dependent postprandial glucose [31]. TFE3 enhances exercise tolerance in mice by increasing glucose metabolism in muscle cells. Genes associated with glucose metabolism, particularly glucose transporter 4, HKII, and glycogen synthase, were significantly increased in muscles overexpressing TFE3, particularly in the soleus, quadriceps femoris, gastrocnemius, and tibialis anterior [32]. Glucose transporter 4 is a key molecule for glucose uptake in the muscle, and HKII and glycogen synthetase are key enzymes in glycogen synthesis [33, 34]. This suggests that TFE3 plays a key role in glucose metabolism in muscle cells.

Although the mechanisms of glucose metabolic regulation of TFE3 in skeletal muscle cells and hepatocytes are different, there are a few similarities, such as enhancing cellular sensitivity to insulin. TFE3 plays a substantial role in regulating insulin sensitivity, glucose homeostasis, and skeletal muscle endurance.

### TFE3 fusion proteins in tumor cells can provide a favorable metabolic environment by interfering with circadian rhythm, insulin activity, and lactate metabolism

In NONO-TFE3 translocation renal cell carcinoma (tRCC), the NONO-TFE3 fusion protein can directly inhibit tumor necrosis factor receptor-associated factor 3-interacting protein 2-antisense 1 (TRAF3IP2-AS1) expression. TRAF3IP2-AS1 can inhibit poly(ADP-ribose) polymerase 1 (PARP1) expression by directly binding and recruiting the N6-methyladenine methyltransferase complex. In other words, the NONO-TFE3 fusion protein can increase the expression level of PARP1 by downregulating TRAF3IP2-AS1 expression [35]. Abnormal expression of PARP1, a polymerase involved in metabolic regulation, leads to cell metabolism disorder. The activation of PARP1 reportedly leads to insulin resistance, likely by interfering with glucagon-like peptide 1 signaling to reduce insulin secretion in β-cells [36, 37]. In astrocytes, PARP1 also inhibits the activity and glycolysis flux of glyceraldehyde-3-phosphate dehydrogenase by decomposing NAD^+^ [38]. In addition, PARP1 is involved in the regulation of circadian rhythm, the disturbance of which may increase the risk and consequences of obesity [3, 39, 40]. Therefore, the high energy levels required for the abnormal proliferation of tumor cells in NONO-TFE3 tRCC may be achieved by the abnormal expression of PARP1, when induced by NONO-TFE3 fusion protein.

Alveolar soft part sarcoma (ASPS) is a chromosomal translocated soft tissue malignancy, with etiology that may be associated with t(X;17)(p11.2;q25) translocation of the fusion gene *ASPSCR1-TFE3* [41]. In ASPS, the TFE3 fusion protein can lead to an increase in lactic acid in the tumor microenvironment. Tumor tissue can use endogenously or exogenously increased lactic acid as a metabolic substrate to produce energy, resulting in tumor cell proliferation and angiogenesis, which may be a necessary condition to maintain a high energy consumption state within the tumor [42, 43].

### TFE3 can promote the metabolism of adipocytes

In addition to hepatocytes and muscle cells, TFE3 regulates adipocyte metabolism. White adipose tissue plays an important role in lipid metabolism, and it can store and decompose triacylglycerol. Fatty acids produced by the decomposition of triacylglycerols provide an energy source for other organs. Subsequently, brown adipose tissue uses fatty acids produced by fat decomposition in white adipose tissue to activate the uncoupling protein 1 (UCP1). UCP1 is a mitochondrial inner membrane protein that rapidly burns fat to produce heat and maintain body temperature under cold stimulation [44].

TFE3 can trans-activate the E-box site of *PGC-1α*. PGC-1α can stimulate UCP1 expression in brown adipose tissue during cold shock, thereby accelerating non-shivering thermogenesis. This is the mechanism through which TFE3 protects against cold damage by regulating lipid metabolism [45, 46]. Alternatively, Arany (2008) found that PGC-1α activation enhances the oxidation of fatty acids [47]. Chromatin immunoprecipitation coupled with high-throughput sequencing of pluripotent stem cells revealed that *PGC-1β* is also a TFE3 target gene [48]. TFE3 can bind to the highly conserved first intron of *PGC-1β*, an enhancer region, to directly enhance *PGC-1β* expression, and PGC-1β activation can highly induce browning in white adipose tissue [49]. Notably, a compensatory increase in PGC-1α expression can be found in adipose tissue after PGC-1β loss. Although partial complementarity of PGC-1α and PGC-1β functions in adipose tissue may exist, the reversal of the phenotype caused by PGC-1β loss remains insufficient [49]. Therefore, the activation of PGC-1β is necessary and sufficient for the browning of white adipose tissue. However, other researchers had found that when transgenic mice with fat-specific overexpression of TFE3 were stimulated by cold, their body temperature was lower than that of the WT group. Luciferase analysis revealed that TFE3 inhibits adipogenesis in white adipose tissue by reducing the gene expression of its rate-limiting enzyme and significantly inhibits the expression of thermogenesis genes such as *UCP1* and *UCP2* [19]. Abnormal expression of TFE3 can lead to abnormal fat browning. However, the specific regulatory mechanism remains elusive.

## TFE3 regulates cell metabolism via mitochondria

### TFE3 regulates organismal metabolism by adjusting mitochondrial dynamics and the mitochondrial respiratory chain

Mitochondria play a central role in cellular energy metabolism [50, 51]. Mitochondria can convert organic substances, such as glucose and fat, into adenosine triphosphate (ATP) through respiration. Therefore, mitochondria are also known as the “energy factory” [52]. Numerous studies have revealed that TFE3 is closely related to mitochondrial respiratory chain formation, cellular respiration, and mitophagy. TFE3 indirectly affects cellular metabolism through mitochondrial regulation [4, 53].

After observing the muscle and liver of *Tfe3* KO mice using an electron microscope, a group of numerous mitochondria with abnormal morphology was identified. The size and quantity of mitochondria were greater in the muscle. Mitochondria in liver cells exhibited round and large outlines regardless of nutritional status; however, no significant change was observed in the number of mitochondria in liver cells compared with that in normal cells [4]. These results indicate that TFE3 plays an important role in mitochondrial dynamics. To explore the specific mechanism of changes in mitochondrial size and quantity, Pastore et al. analyzed the published chromatin immunoprecipitation sequencing data of TFE3 in mouse embryonic stem cells. They found that 136 mitochondria-related genes targeted by TFE3 were highly enriched in the fission pathway. Among them, the most prevalent is the mitochondrial fission receptor Fis1. In addition, Drp1 is a cytoplasmic protein that accumulates around the mitochondrial outer membrane to form a loop. Fis1 and Drp1 are key regulators of mitochondrial fission [4, 48], and they can interact to promote mitochondrial division [54, 55]. In addition, the overexpression of TFE3 in the liver strengthens the expression of genes involved in mitochondrial dynamics (e.g., *Fis1, Drp1, OPA1, Mfn1*, and *Mfn2*). These two findings demonstrate that TFE3 can directly bind to the *Fis1* promoter to regulate mitochondrial cleavage. Simultaneously, *Pgc-1α* is closely related to the occurrence and remodeling of mitochondria, and its promoter can also be bound by TFE3 to mediate transcriptional activation [4, 56, 57].

The overexpression of TFE3 in hepatocytes also leads to a remarkable increase in the content of protease complexes (e.g., complexes I, II, and III) in the oxidized respiratory chain of mitochondria. In contrast, a decrease in the protein content of the protease complex was observed. A remarkable decrease in membrane potential and oxygen consumption rate was also observed in the hepatocytes of *Tfe3* KO mice, which indicated that the loss of *Tfe3* severely inhibited the oxidative function of mitochondria [4]. These results suggest that TFE3 could maintain normal metabolic activity within an organism by promoting the oxidative decomposition of chemicals to produce ATP.

### TFE3 hinders metabolic disease progression by activating alternative mitophagy

Mitophagy is a process through which autophagosomes wrap damaged mitochondria and subsequently initiate autophagy clearance [58]. Mitophagy plays an important role in mitochondrial quality control and is necessary for maintaining the normal metabolic capacity of mitochondria [59]. Mitophagy involves two pathways: the Atg5/Atg7-dependent traditional pathway and the small GTPase Ras-related protein 9 (Rab9)-dependent alternative pathway [60, 61]. In a model of obese cardiomyopathy resulting from a high-fat diet, acute-phase mitochondrial injury in cardiomyocytes can be cleared via the conventional mitophagy pathway [62]. This pathway is typically inhibited during the chronic phase of this model, and alternative mitophagy pathway activity increases [63, 64]. Evidently, TFE3 can lead to alternative mitophagy by binding to the *Rab9* promoter. In cases of chronic mitochondrial injury to cardiomyocytes, owing to a late high-fat diet, alternative mitophagy occurs because of the upregulation of TFE3 rather than the increased affinity of TFE3 to the *Rab9* promoter. Meanwhile, activating the alternative mitophagy pathway can salvage mitochondrial damage caused by the reduced activity of the conventional mitophagy pathway [65]. This also suggests that indirectly or directly increasing TFE3 content by recombinant human TGF-β1 or by adenovirus expressing human TFE3 may delay the progression of metabolic diseases caused by the lack of traditional mitochondrial autophagy [4, 66]. This idea provides a novel direction for the treatment of mitochondrial disease.

### TFE3 enhances the metabolism of cancer cells by altering mitochondrial distribution and function

Kidney cancer is a metabolic disease, and most genes (e.g., *TFE3*, *TFEB*, *FLCN*, *MITF*) involved in this cancer can affect the tricarboxylic acid cycle of cells, which primarily occurs within mitochondria [67, 68].

The TFE3 fusion protein in tRCC promotes mitochondrial division and enriches mitochondria at the cell front edge by promoting Fis1 and Drp1 expression. These alterations can supply more local ATP to cancer cells and F-actin network growth, thereby increasing the metabolic capacity of cancer cells and providing sufficient energy for further migration and invasion [69]. The TFE3 fusion protein can also accelerate the mitochondrial generation and energy production by activating the PGC-1α and NRF1 pathways, thereby providing more energy for the proliferation of cancer cells [57, 70, 71]. In PRCC-TFE3 tRCC, the TFE3 fusion protein reduces the generation of reactive oxygen species, which can damage mitochondria. In contrast, the TFE3 fusion protein can promote pink1-PRKN-dependent mitophagy through the activation of the target gene E3 ubiquitin ligase PRKN. Mitophagy enables the release of cyclin from damaged mitochondria to protect cells from apoptosis caused by reactive oxygen species. These alterations provide a stable metabolic environment for the infiltration and proliferation of cancer cells [72].

## TFE3 regulates cell metabolism by enhancing lysosomal activity and activating autophagy

### TFE3 indirectly regulates metabolism by enhancing lysosomal activity

Previously, lysosomes were considered to participate in the degradation and recycling of cellular waste products. However, there is now compelling evidence that lysosomes have broader functions, such as involvement in cellular metabolism [73]. TFE3 can enhance lysosomal activity by promoting lysosome biosynthesis and exocytosis to regulate metabolism [5, 74].

**Fig. 2:**
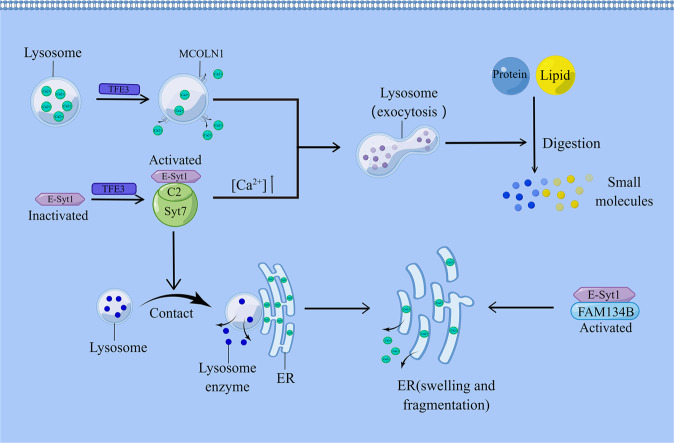
Schematic of TFE3 regulating lysosomal activity and Ca^2+^ concentration. TFE3 upregulates and activates MCOLN1, releasing large amounts of Ca^2+^. E-Syt1, which is activated by TFE3, forms a heterodimer with the C2 domain of Syt7, triggering lysosomal exocytosis. Both pathways can increase lysosomal activity and enhance lipid and protein digestion. Alternatively, the heterodimer can mediate lysosome contact with the ER, with the subsequent release of lysosomal enzymes to fragment the ER and release Ca^2+^ from the ER. E-Syt1 can also bind to FAM134B, and activated FAM134B can mediate ER swelling and fragmentation, releasing Ca^2+^.

The exocytosis of lysosomes is related to the intracellular free Ca^2+^ concentration [75]. Currently, there are two key mechanisms for how TFE3 increases Ca^2+^ concentrations in response to a stress state. Alternatively, TFE3 can directly bind to the homologous region of the coordinated lysosomal expression and regulatory (CLEAR) elements in the E-Syt1 promoter of HeLa cells, which is a 10 bp DNA motif (5’-GTCACGTGAC-3’) located -1008 bp from the transcription start point. Activated E-Syt1 forms a heterodimer with the C2 domain of synaptotagmin-7 (Syt7), a Ca^2+^ sensor on Syt7 that can trigger lysosomal exocytosis by sensing elevated Ca^2+^ concentrations within the cytosol. The formed dimers mediate contact between lysosomes and the ER. Subsequently, enzymes released from lysosomes cause nearby ER fragmentation, and Ca^2+^ is released from the fragmented ER [42, 74]. ESyt1 can also bind to FAM134B, and activated FAM134B can mediate ER swelling and fragmentation, which releases large amounts of Ca^2+^ [76, 77]. In contrast, Martina et al found that the overexpression of TFE3 in ARPE-19 cells leads to increased expression of the lysosomal Ca^2+^ release channel MCOLN1, which could mediate Ca^2+^ release. Furthermore, TFE3 can bind CLEAR elements in the promoter region of several lysosomal generation-related genes to induce lysosome biogenesis [5]. Regardless of the pathway TFE3 passes, it eventually leads to increased lysosomal production and exocytosis, resulting in an increased lysosomal activity. Activated lysosomes can enhance the digestion of lipids and proteins, which are subsequently converted to small molecules, exported to the cytoplasm, and immediately used for daily metabolic activities of the cells [78–81] (Fig. 2).

### TFE3 regulates metabolism by directly regulating autophagy depending on the nutritional status of the cell

Several studies have revealed that TFE3 is a key regulator of autophagy and that autophagy is closely associated with the metabolic regulation of cells [82, 83]. Metabolism regulation via autophagy is primarily achieved through the mammalian target of the rapamycin 1(mTOR1) pathway [84].

**Fig. 3:**
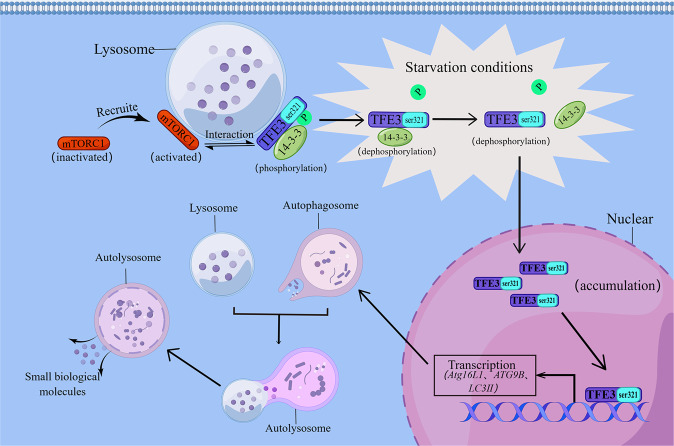
Mechanism of TFE3 affects autophagy to regulate metabolism in different nutritional states. In the normal state, mTORC1 is recruited to the lysosome surface and is activated to facilitate synthesis and inhibit autophagy. The active mTORC1 phosphorylates TFE3 on serine 321 and generates the 14-3-3-binding site. When starved, dephosphorylated ser321 separates TFE3 from the 14-3-3 sites, causing TFE3 accumulation in the nucleus, resulting in the upregulated expression of corresponding target genes (e.g., *Atg16L1*, *ATG9B*, and *LC3-II*). Subsequently, autophagosomes phagocytose intracellular material and fuse with the lysosome to form autolysosomes that degrade phagocytic substances into small biomolecules.

Martina et al. found that in a fully fed human retinal epithelial pigment cell line ARPE-19, mTORC1 is recruited to the lysosomal surface and activated to promote anabolic processes, such as protein synthesis and nutrient storage, and to inhibit autophagy. TFE3 is recruited to lysosomes by interacting with Rag heterodimers (RagB_GTP_/RagD_GDP_) in an active conformation. If the Rag-binding domain on TFE3 is mutated (TFE3-S112A/R113A), TFE3 will fall off the lysosome and gather in the nucleus, even under adequate nutrition conditions. Following active mTORC1 phosphorylation of TFE3 on serine 321, a binding site for 14-3-3, a cytoplasmic chaperone that isolates TFE3 in the cytoplasm is produced. In contrast, under starvation conditions, RagGTPases and mTORC1 are co-inactivated, and ser321 dephosphorylation prevents the TFE3 protein from interacting with 14-3-3. This leads to a rapid accumulation of TFE3 in the nucleus. After TFE3 enters the nucleus, the corresponding target genes that play key roles in autophagosome formation (*Atg16L1*, *ATG9B*, *LC3-II*, *GABARAPL1*, and *WIPI1*) and its degradation (*UVRAG*) are upregulated [5, 85]. Autophagosomes engulf large amounts of intracellular substances and fuse with lysosomes to form autolysosomes. Subsequently, autolysosomes rapidly degrade phagocytic substances into small biological molecules that are transported to the cytoplasm through specific lysosomal receptors for cell metabolism and reuse (Fig. 3) [86, 87]. Through the activation of TFE3-induced autophagy, cells can use sporadic energy to survive, even in a state of nutrient deficiency. This pathway provides an emergency metabolic pathway for cells to utilize new energy sources under extreme environmental conditions.

### TFE3 plays different roles in Xp11tRCC metabolism at different periods via the activated autophagy pathway

Xp11 tRCC is a distinct subtype of renal cell carcinoma characterized by the presence of multiple chromosomal translocations involving *TFE3* on the Xp11.2 chromosome, resulting in sustained overexpression of TFE3. The three most common Xp11 tRCC fusion sites are PRCC, ASPL, and SFPQ [88, 89]. TFE3 fusion proteins constantly shuttle between cytoplasm and nucleus at a greater rate than wild-type TFE3 proteins; therefore, there are more fusion TFE3 proteins than wild-type TFE3 in nuclei. Thus, TFE3 fusion proteins regulate the metabolism of cancer cells through autophagy differently from TFE3 in normal cells [90]. Its mechanism is not entirely dependent on the nutritional status of the cell and is not identical in the early and late stages of cancer development [91–94].

In the early stages of Xp11 tRCC, autophagy activated by TFE3 first suppresses and subsequently promotes the metabolism of cancer cells. The TFE3 fusion protein can directly bind to the promoter of *p21*^*WAF1/CIP1*^ in HEK293/T-REx/PRCCTFE3 cells, causing cell cycle arrest and senescence by hindering the transformation of the G1 and G2 phases to the S phase, thereby ensuring reduced metabolic growth levels in mutant cells [90, 92]. Brady et al. compared p53 activation and stabilization in wild-type and TFEB/TFE3 double-knockout RAW264 cells and found that TFE3 and TFEB improved p53 stability and protein levels through feedforward and feedback pathways, thereby increasing the expression of multiple DNA damage-response transcripts to repair damaged DNA fragmentation. When DNA repair is complete, TFE3 returns cells to normal metabolic growth levels by promoting autophagy and restarting the cell cycle [93, 95].

In the late stage of Xp11tRCC, autophagy activated by TFE3 plays a major role in promoting cancer cell metabolism [91]. Autophagy and mTOR signaling pathways regulate cell metabolism during cell starvation and adequate nutritional conditions, respectively. TFE3 primarily enhances cell metabolism [82, 83, 96]. In general, TFE3 activates autophagy only when the mTOR signaling pathway is inactivated [5]. However, in ACHN cells, the ASPL–TFE3 fusion protein always remains in the nucleus and maintains the autophagy pathway in tumor cells, regardless of whether the cells are in a nutrient-deficient environment [97]. The deletion of the TFE3 Rag-binding domain, which is the binding site of TFE3 and active RagGTPases, prevents the phosphorylation of TFE3 by mTORC1 to produce 14-3-3 binding sites and remains in the cytoplasm in carcinoma cells [5]. This region is missing from the TFE3 fusion protein of both UOK109 and UOK120 cells [98]. Numerous studies have revealed that TFE3 activation is regulated by the mTOR pathway [98]. However, recent studies have found that TFE3 may regulate mTOR pathway activity. In RP-R07 cells, TFE3 binds to its downstream target gene, *IRS-1*, which subsequently causes increased phosphorylation of the S6 ribosomal protein and 4E binding protein-1. Phosphorylation of the S6 ribosomal proteins and 4E binding protein-1 can represent the activation levels of mTOR signaling, as they occur at the end of mTOR signaling. This shows that TFE3 can regulate the mTOR signaling pathway through a feedback loop mechanism [99, 100]. Similarly, Di Malta et al. found that TFE3 directly regulates RagD expression in HeLa cells, thereby controlling the recruitment of mTORC1 to lysosomes [101]. This provides a new concept for the simultaneous activation of autophagy and the mTOR signaling pathway in tRCC.

The *TFE3* fusion gene can continuously provide the metabolic raw material for cells by activating autophagy. Simultaneously, the activation of the mTOR signaling pathway can better use autophagy decomposition products to promote the synthesis of various substances for the metabolism of tumor cells. The coactivation of autophagy and mTOR signaling pathways is mutually dependent on the regulation of the metabolism of tumor cells [74]. TFE3 enables tumor cells to obtain sufficient energy by activating the autophagy and mTOR signaling pathways. Therefore, tumor cells can rapidly grow and proliferate in a state of both rich and poor nutrition.

## Conclusions and perspectives

TFE3 plays an important role in biological metabolism. It participates in lysosomal biogenesis, cancer progression, autophagy induction, and glucose and lipid metabolism. In this review, we have gained insights into the specific mechanisms and roles of TFE3 in cell metabolism (Table 1).

**Table 1:** Role of TFE3 in different cells.

Location	Function	Related molecules	PMID
Hepatocyte	Regulates insulin and glucose metabolism	IRS-2, FOXO1, SREBP-1c, PGC-1α, and HKII	16327801, 23269357
Skeletal muscle cell	Regulates glucose metabolism	Glucose transporter 4, HKII, and glycogen synthetase	22297304, 23899560
Tumor cell	Interferes with circadian rhythm, insulin activity, and lactate metabolism	TRAF3IP2-AS1, and PARP1	21458523, 22921416, 14523042, 14715917
Adipocyte	Regulates lipid metabolism	UCP1, PGC-1α, and PGC-1β	18782618, 23885019, 10871882

TFE3 can directly regulate body metabolism and indirectly regulate the function of organelles (such as lysosomes and mitochondria) and autophagy activity. Several studies suggest that some metabolic diseases are associated with the abnormal expression of TFE3, such as diabetes mellitus, obesity-related cardiomyopathy, and ASPS. Moreover, an alteration in TFE3 expression can also alter the developmental trend of such diseases [4, 42, 43, 65]. Therefore, in the treatment of metabolic disease, the regulation of TFE3 expression in related tissues or cells may produce remarkable therapeutic effects.

We have summarized a few research directions for the screening of effective interventions for metabolic diseases. However, some questions regarding metabolic regulation by TFE3 remain controversial. The traditional view is that TFE3 overexpression can promote fat browning in adipocytes, accelerate the metabolism of adipose tissue, and manage cold stimulation. In contrast, some studies have revealed that transgenic mice overexpressing TFE3 in adipocytes have low body temperatures in cold environments. Therefore, there is no consensus on whether TFE3 promotes or inhibits fat browning. We assumed that the regulation of fat browning by TFE3 may be related to the level of TFE3 expression, although the specific mechanisms need to be studied further. In addition, TFE3 fusion proteins in cancer cells can perform several metabolic regulatory functions that are not present in TFE3 itself, such as the reverse regulation of mTOR pathway activity. This also shows that TFE3 can play a new role after binding to other proteins, which is crucial for our research on the pathogenesis of cancer and the development of new drugs. Therefore, further investigation of the specific mechanisms underlying the role of TFE3 in body metabolism is warranted. We can explore the specific mechanism of the interaction between TFE3 and related molecules or proteins by making it either physiologically expressed or abnormally expressed. More in-depth screening of the upstream and downstream pathways of TFE3 is required to further clarify the relevant signal pathways of TFE3 in regulating biological metabolism.

In conclusion, TFE3 plays an important role in organismal metabolic regulation. However, the specific mechanisms of TFE3 in metabolic processes require further research. Systematic approaches, such as transcriptomics, proteomics, and metabolomics, coupled with systems biology, are particularly important for identifying all the components of TFE3, TFE3 fusion proteins, and the upstream and downstream pathways in which they participate.

## Data Availability

All data that support the findings of this study are available from the corresponding author upon reasonable request.
